# An Analysis of Computed Tomography Diagnostic Reference Levels in India Compared to Other Countries

**DOI:** 10.3390/diagnostics14151585

**Published:** 2024-07-23

**Authors:** Maajid Mohi Ud Din Malik, Mansour Alqahtani, Ibrahim Hadadi, Abdullah G. M. AlQhtani, Abdullah Alqarni

**Affiliations:** 1 Dr. D.Y. Patil School of Allied Health Sciences, Dr. D.Y. Patil Vidyapeeth, Sant Tukaram Nagar, Pimpri, Pune 411018, MH, India; majidmalik343@gmail.com; 2 Department of Radiological Sciences, College of Applied Medical Sciences, Najran University, Najran 61441, Saudi Arabia; mmalqahtane@nu.edu.sa; 3 Department of Radiological Sciences, College of Applied Medical Sciences, King Khalid University, Abha 61421, Saudi Arabia; 4 Radiology and Medical Imaging Department, College of Applied Medical Sciences, Prince Sattam Bin Abdulaziz University, Alkharj 11942, Saudi Arabia; ag.alqahtani@psau.edu.sa (A.G.M.A.); aa.alqarni@psau.edu.sa (A.A.)

**Keywords:** computed tomography, diagnostic reference level, dose-length product, CT dose index

## Abstract

Computed Tomography (CT) is vital for diagnosing and monitoring medical conditions. However, increased usage raises concerns about patient radiation exposure. Diagnostic Reference Levels (DRLs) aim to minimize radiation doses in CT imaging. This study examines CT DRLs in India compared to other countries to identify optimization opportunities. A literature review was conducted to gather data from published studies, guidelines, and regulatory authorities. Findings show significant international variations in CT DRLs, with differences up to 50%. In India, DRLs also vary significantly across states. For head CT exams, Indian DRLs are generally 20–30% lower than international standards (27–47 mGy vs. 60 mGy). Conversely, for abdominal CT scans, Indian DRLs are 10–15% higher (12–16 mGy vs. 13 mGy). Factors influencing DRL variations include equipment differences, imaging protocols, patient demographics, and regulatory conditions. Dose-optimization techniques like automatic exposure control and iterative reconstruction can reduce radiation exposure by 25–60% while maintaining image quality. Comparative data highlight best practices, such as the United Kingdom’s 30% reduction in CT doses from 1984 to 1995 via DRL implementation. This study suggests that adopting similar practices in India could reduce radiation doses by 20–40% for common CT procedures, promoting responsible CT usage and minimizing patient exposure.

## 1. Introduction

Using X-rays and computational reconstruction techniques, computed tomography (CT) creates three-dimensional images of an individual’s internal components. Since Godfrey Hounsfield first developed it in 1969, it has found increasing use in the medical field. Before 2008, over 3.6 million annual CT scans were performed as part of diagnostic radiology exams worldwide [1]. Medical radiology was predicted to contribute 60–70% of projected doses, with CT procedures contributing 25% of all diagnostic-examination doses. CT-examination medical exposures accounted for 34% of the total dose. Due to the disproportionately high dosage nature of CT examinations, they account for almost 50% of the total radiation exposure in various countries, notably 68% in the United Kingdom [2]. According to AAPM report no. 204, 80 million CT scans were performed in the US annually. In 2006, the United States experienced seven times the medical exposure seen in the 1980s [3]. CT procedures accounted for 12% of all medical imaging performed and over 50% of the total radiation dose received by the American population. Collectively, the effective dose of the world’s population from CT medical exams is between 30 and 50% of examinations, and the patient dose they entail has increased dramatically over the past decade. In contrast, traditional radiography doses have decreased by about 30%. As a result, the overall medical radiation dosage received by the population each year and the considerable dose received per examination are rising. 

This narrative review was conducted through a comprehensive search of peer-reviewed literature, regulatory guidelines, and national reports on CT DRLs. Our objectives were to (1) analyse CT DRLs in India and compare them with international standards, (2) identify factors influencing DRL variations, and (3) explore potential strategies for optimizing CT radiation doses in the Indian healthcare context (Table 1).

In radiography, a higher X-ray exposure is associated with improved image quality, which is necessary for acceptable diagnostic reasons. Similarly, CT scans use X-radiation to achieve images with sufficient diagnostic detail. Tissue reactions (such as erythema, infertility, hair loss, cataracts, etc.) and stochastic consequences (such as cancer and genetic impacts) have been linked to high-dose ionizing radiation exposure. The increased likelihood of cancer is a result of the patient’s exposure to higher CT dosages. After obtaining a medical diagnosis, this is one of the most significant sources of anxiety for the patient [2]. As a result, its clinical uses necessitate vigilant examination of exam conditions, not to mention the optimization of patient dose and image quality. Therefore, CT examinations should be performed at dosages close to as low as possible, consistent with the desired diagnostic accuracy. In order to effectively promote and facilitate the execution of patient dose minimization throughout the imaging process, the cooperation of experts is crucial. Automatic exposure control, bow-tie/beam shaping filters, and various CT parameters, including kVp settings, filtration, and tube-current modulation, play crucial roles in determining patient dose for abdominal CT, and usage of the anti-scatter grid, tube-current modulation, selective in-plane shielding [5], and thyroid and breast shields are the most important CT parameters that determine the amount of dosage received by a patient [6,7]. Different CT scanners have unique techniques for exposure control because of variances in equipment and user expertise. Automatic exposure controls, cautious patient referral criteria, choosing appropriate scan parameters, and conducting a thorough assessment of the protocol are all highly effective approaches that can be utilized to successfully lower the potentially high dose of CT examinations. These methods, generally expressed in percentage terms, are highly applicable for reducing the amount of radiation exposure received during cancer treatment. The radiation-protection concept must be closely adhered to in order to adjust all these CT parameters during diagnosis in such a way that they supply the diagnostic information needed without losing the quality of the image. Justification for each examination and protection maximisation are the cornerstones of medical exposure, and these ideas will be briefly discussed below in relation to CT medical diagnosis. 

Techniques of optimization strategies, together with an estimate of the reduction in CT dose provided by various optimization techniques, have been developed and implemented in CT imaging to reduce patient radiation dose while maintaining diagnostic image quality. Here are some common strategies, along with their approximate reduction in CT dose:

### 1.1. Automatic Exposure Control (AEC)

AEC adjusts the tube current based on the patient’s anatomy and size, ensuring that the appropriate radiation dose is delivered only where needed. This technique can lead to approximately 25–50% dose reductions compared to fixed tube current scans [8].

### 1.2. Tube Voltage Selection (kVp Optimization)

Lowering the tube voltage (kVp) while maintaining image quality can reduce patient dose. A reduction of 10 kVp can yield dose reductions of around 20–30% [9].

### 1.3. Iterative Reconstruction Algorithms

Iterative reconstruction algorithms improve image quality by reducing noise and artefacts, allowing for lower radiation dose settings. These algorithms can achieve dose reductions of approximately 30–60% compared to traditional filtered back projection [10].

### 1.4. Dose Modulation

Dose modulation techniques, such as angular and z-axis tube current modulation, dynamically adjust the tube current during the scan based on the patient’s anatomy. Depending on the implementation and patient characteristics, a 20–50% reduction can be achieved [11].

### 1.5. Pitch Adjustment

Increasing the pitch value (table feed per rotation) can reduce the overlapping data acquired, leading to 10–40% dose reductions. However, higher pitch values may compromise image quality in certain cases [8].

### 1.6. Organ-Based Dose Reduction

Specific protocols can tailor the radiation dose to different organs or regions of interest. By reducing the dose to non-diagnostic areas, the overall patient dose can be lowered by approximately 10–30%.

### 1.7. Dose Tracking and Monitoring

Regular dose tracking and monitoring practices enable facilities to identify outliers and implement corrective actions to maintain dose levels within acceptable ranges. Such measures can lead to continuous improvements and an approximately 10–20% dose reduction [12].

### 1.8. Paediatric-Specific Protocols

Implementing protocols optimized for paediatric patients can significantly reduce their radiation exposure. Age- and size-specific settings can achieve dose reductions of 50% or more compared to adult protocols.

It is essential to note that the degree of dose reduction achieved with each technique may vary depending on the specific CT equipment, imaging protocols, patient population, and clinical requirements. Moreover, achieving lower radiation doses should always balance maintaining sufficient image quality for accurate diagnosis. Regular quality assurance and monitoring are crucial to ensure that dose-optimization strategies are effective and do not compromise patient care [13].

### 1.9. Radiation Protection in Computed Tomography [14]

The radiation-protection principles in Computed Tomography (CT) are based on the ALARA principle, “As Low As Reasonably Achievable”. This principle emphasizes minimizing patient radiation exposure while maintaining diagnostic image quality. The goal is to balance obtaining the necessary medical information with reducing radiation risks to the lowest possible level. The following are the key radiation-protection principles in CT:

#### 1.9.1. Justification

CT scans should only be performed when there is a clear clinical indication and the information obtained is essential for patient management. The benefits of the CT scan must outweigh the potential risks associated with radiation exposure.

#### 1.9.2. Optimization

CT protocols need to be improved so that the requisite diagnostic picture quality can be obtained with the minimum amount of humanly achievable radiation exposure. CT scans can be optimised by the use of a variety of techniques, such as changing the tube current (mA) and tube voltage (kVp) and employing iterative reconstruction algorithms. 

#### 1.9.3. Automatic Exposure Control (AEC) 

AEC systems adjust the tube current based on the patient’s size and anatomy, tailoring the radiation dose to individual patients. This helps to avoid overexposure to radiation for smaller patients and maintains image quality for larger patients.

#### 1.9.4. Patient Dose Monitoring and Dose Alerts

Regularly monitoring patient radiation doses and implementing dose alerts for outlier cases helps identify instances where dose levels may be higher than expected. This enables corrective actions to be taken and contributes to continuous improvement in radiation protection.

#### 1.9.5. Paediatric and High-Risk Groups

Special consideration should be given to paediatric patients and other vulnerable groups, such as pregnant women, to minimize their radiation exposure. Protocols tailored to these groups should be used whenever possible.

#### 1.9.6. Education and Training

Proper education and training of CT technologists and radiologists are crucial to ensure they understand radiation-protection principles and can implement optimized imaging techniques.

#### 1.9.7. Collimation and Shielding

Proper collimation ensures that only the necessary anatomy is exposed to radiation, reducing unnecessary exposure of adjacent tissues. Appropriate shielding can also protect sensitive organs from radiation during CT scans.

#### 1.9.8. Quality Assurance and Quality Control

Regular quality assurance testing of CT equipment and ongoing quality-control measures help ensure that the imaging systems function correctly and produce accurate images at optimized radiation doses.

#### 1.9.9. ALARA Culture

Creating a culture of radiation safety and awareness in healthcare facilities is vital. All staff involved in CT imaging should be aware of the ALARA principle and actively participate in radiation protection practices.

By adhering to these radiation protection principles, healthcare facilities can maintain patient safety and ensure that CT scans remain an effective and valuable diagnostic tool while minimizing potential radiation-related risks.

### 1.10. Justification of Medical Exposure in CT 

Unless doomed to failure, any radiation-protection concept worth its salt will account for clinical practice in the actual world. “Any use of radiation for medical practice should be justified”, states the ICRP’s guiding concept [15]. The IAEA emphasizes that “the basic purpose of medical exposure is to do better than hurt the patient” [16]. Justification is defined thus: “Decisions introducing a practice shall be justified in the sense that such decisions shall be taken with the intent to ensure that the individual or societal benefit resulting from the practice outweighs the health detriment that it may cause” [17]. This definition comes from the European Commission (E.C.) council directive 2013/59/Euroatom. That means X-ray exams are not used unless they are medically necessary. This means referring doctors and radiologists should know how doses might be lowered by adjusting technical settings. Patient radiation exposure can be reduced by more than 50% with careful choice of technical parameters, sound quality-control procedures, and effective application of DRLs [18]. Establishing and demonstrating norms of acceptable practice, analysing harms for justification, and risk assessment are the primary motivations for defining patient doses in diagnostic medical imaging [19]. 

### 1.11. Optimization of Medical Examination in CT

After justification has been made, further optimization of protection is required to strike a healthy balance between patient exposure and crucial diagnostic information. “Registrants, licensees, and radiological medical practitioners shall ensure that protection and safety are optimized for each medical exposure” [20], states the IAEA basic safety standard (BSS). The principle of optimization states, “Exposures should be kept as low as reasonably attainable (ALARA), taking societal and economic impacts into account”. This includes minimizing the likelihood of exposure, the number of persons exposed, and the volume of individual doses. Therefore, CT centres should guarantee that exams are performed with the lowest possible patient dose without sacrificing clinical image quality. The primary goal of the optimization is to avoid or minimize future exposures to obtain the highest possible level of patient protection and to provide sufficient investigative data. When optimizing patient dose in diagnostic radiology, DRLs are crucial. 

### 1.12. Quantities for CT Dosimetry 

Whenever a patient undergoes a radiological test, the potential benefits are expected to outweigh the potential risks. Therefore, medical professionals must comprehend the gravity of radiation hazards connected with radiological tests and how these risks differ with patient age and sex [21]. Because computed tomography (CT) imaging typically involves substantially higher radiation doses than traditional radiography (X-ray) or fluoroscopy (fluoroscopic imaging), patients must be informed about the risks involved. Although CT only made up 17% of all diagnostic exams performed in 2006, it was responsible for roughly 50% of the population’s exposure to medical imaging. CT dose (CTDI_VOL_) quantifies the radiation exposure during a CT exam independent of scan duration. The DLP and the CTDI_VOL_ are metrics that help technologists make decisions during CT scans (DLP). The DLP is calculated by multiplying the CT dose equivalent volume by the scan length to measure the overall dose received during a CT scan. However, how much radiation a patient is exposed to varies, depending on factors including their body type and the CT procedure being performed.

### 1.13. CT Dose Descriptors

#### 1.13.1. Dose-Length Product (DLP)

The radiation emitted by a CT tube is expressed as a dose-length product (DLP) in milligrays per centimetre. DLP considers the radiation source’s z-axis length (the patient’s long axis) [22,23]. CTDIVOL is similar to the volume CT dose index. However, it only measures the dose through a single phantom slice.

DLP = (CTDIVOL) × (length of scan, cm)

The unit to measure DLP is mGy*cm.

#### 1.13.2. Computed Tomography Dose Index (CTDI)

A standardized measure called the CT dose index (CTDI) was introduced to facilitate the comparison of radiation dose output among different CT scanners. Initially, CTDI was measured over a 100 mm long ionization chamber (CTDI100), along with CTDIW for weighted measurements. However, modern helical scanners commonly utilize CTDI_VOL_ instead of CTDI100 and CTDIW [24]. CTDI_VOL_ allows for a more accurate and comprehensive radiation dose assessment in volumetric CT acquisitions [24,25]. While the CTDIVOL (or its derivative, the DLP) is commonly displayed on consoles and included in DICOM images, it is essential to note that it does not directly represent the actual absorbed or effective dose for the patient. Therefore, it should not be solely relied upon as a precise measure of patient radiation dose. Instead, these parameters should be used as indicators of the radiation output of the imaging system, enabling comparisons between different systems. Additional factors and dose-estimation techniques should be considered [26] to assess patient dose accurately. In computed tomography (CT) imaging, the radiation dose to a patient is measured using a metric called the Computed Tomography Dose Index (CTDI). It estimates the radiation dose within a limited area of the patient’s body, usually the area being examined. Assessing the CT scanner’s dose to a single slice or a set of contiguous slices is where CTDI shines. Two primary forms of CTDI are in widespread use today: 

##### CTDIvol (CTDI Volume)

- CTDIvol is the most frequently used variant measured in milligray (mGy) units.
- It represents the average radiation dose within a specific region of the patient’s body, usually a cylindrical phantom or a region of interest (ROI).
- CTDIvol is calculated by measuring the radiation dose along the central axis of the phantom or ROI and then averaging it over multiple scan acquisitions.
- The formula for CTDIVOL calculation involves integrating the radiation dose profile across the scan length and dividing it by the nominal slice thickness.
- CTDIvol estimates the radiation-dose-per-unit length along the scan axis and helps compare radiation doses between different CT scanners and protocols.

##### CTDIW (CTDI Weighted)

- CTDIW is an older variant of CTDI that was commonly used before CTDIvol became more prevalent.
- CTDIW is also measured in milligrays (mGy) and represents the average radiation dose within the central region of a phantom or ROI.
- It is calculated by measuring the radiation dose at specific points along the central axis of the phantom or ROI and taking their weighted average.
- The weighting factors used in CTDIW calculation consider the shape of the radiation-dose profile and provide a more accurate representation of the dose distribution.
- While CTDIW is less commonly reported nowadays, it is sometimes used for legacy purposes or when comparing radiation doses from older CT scanners.

### 1.14. DRLS in the International Context 

Clinically, diagnostic radiology aims to get high-quality images that provide sufficient diagnostic clues and shield a patient from unnecessary radiation exposure for the medical purpose of an X-ray. Therefore, applying DRLs in clinical practice is crucial for optimizing a patient’s dose. Thus, in 1996, the International Commission on Radiological Protection (ICRP) proposed the idea of DRL with the primary goal of optimizing patient dose. The concept of DRL began as practical guidance in 2001 and has since expanded into later iterations. According to ICRP 2007, DRLs are “applied using one of the concepts of optimization of protection in medical exposure”. In many imaging modalities, DRLs are an efficient tool that helps optimize patient protection during medical exposure for diagnostic and interventional procedures [27]. DRLs provide a means by which measured doses can be compared to standards established by local, regional, or national dose data composition [27]. Diagnostic Reference Levels (DRLs) are typically defined for a “standard-sized” patient, often considered to be an adult weighing approximately 70 kg. This standardization allows for meaningful comparisons across different facilities and regions. However, it is important to note that individual patient doses may vary based on actual patient size and other factors. The use of a standard patient size in DRL definitions helps to establish a consistent baseline for dose-optimization efforts while acknowledging the need for individualized approaches in clinical practice.

Setting dosage levels, establishing the DRL values and units, standardizing mechanisms for measuring radiation dose, accumulating aggregate data, and creating approaches to using DRLs are all part of the methodology behind developing DRLs [25]. Data on patient doses, based on the median (middle) of the data collected from participating health centres, are acquired as part of establishing DRL. Several IAEA safety documents emphasize the importance of DRL. For instance, “DRL delivers an upper-level guideline of patient dose which enable facility inspection if the patient dose is unwantedly exceeded”, as written in one of its 2001 progressing series publications. “The registrant and licensee shall provide developed DRLs to govern proper protection, safety, and optimization of patient dose”, reads another provision of the IAEA BSS. Patients are better protected and safer when their dose is kept below the stipulated DRL while maintaining clinically acceptable image quality. DRL is a therapeutic dosage limit being investigated as a means of preventing overdosing in patients. When the patient dose is consistently above the relevant DRLs, when the typical dose is falling significantly below the proper DRL, when the exposures are not providing useful diagnostic information, or when the diagnosis is not yielding the expected medical benefit to the patient, the facility must revise its protocol to lower the mean patient dose reasonably below the previously established DRL. Reducing the dose requires corrective actions taken by official institutions [28]. There is no one-to-one relationship between DRLs and the national radiation dosage limit or dose limitation for medical imaging. As a result, they cannot be used for anything in business or government. Quantities of DRL can be estimated using reference-patient-percentile (such as the 90th, 75th, and 50th) dose distribution. Table 2 shows the DRL of certain nations set at the 75th percentile; empty cells in all tables below show the absence of comparable data. 

Table 3 presents the National Diagnostic Reference Level (NDRL) for CT as it is currently used in several Indian states. As shown, there is considerable variation in DRLs across different regions of India. For instance, the CTDI for head CT ranges from 27 mGy in Kerala to 47 mGy in South India. These variations reflect differences in equipment, protocols, and local practices across the country. Such diversity highlights the need for a standardized approach to establishing and implementing NDRLs in India. 

DRL fluctuates with state-of-the-art equipment and is determined by the imaging procedure. Expert groups play a crucial role in setting DRL standards at the national level. DRLs generate unified evidence on the radiation dose received by the patient, which is important for use in professional judgments. Once again, DRLs are critical for prompt examination when there is an out-of-the-ordinary high dose to patients at nearby sites. DRLs need to be reviewed periodically (every 3–5 years) to enhance leading practises at lower patient doses without sacrificing image quality or care. The necessity of implementing national DRLs to reduce patient radiological doses has not been lost in developed nations, which is why they have done so. The European Union (EU) enacted council directives 97/43 Euratom in 1997, immediately after the ICRP recommended the DRL. The clause stated that “each E.U. member state shall promote to develop and use of DRL for radio-diagnostic examinations” (article 4, sub-article 2) [29]. Article 56 of the cancelled council directives 97/43 Euratom stated, “Member states shall ensure the establishment, frequent review, and use of DRLs for radio-diagnostic exams”. This directive was repealed on 5 December 2013 via the council directives 2013/59/Euratom. Therefore, 23 out of 36 nations have created their own national DRL, as the European Commission (2018) reported [28]. 

Table 4 shows the DRLs for a few E.U. countries. McCullough’s 2010 study from the UK national dosage survey found that DRLs resulted in a 30% decrease in doses (from 1984 to 1995) and a 50% average reduction (from 1985 to 2004). However, 55 emerging African countries adopt a rudimentary method to capitalize on the importance of DRLs to safeguard patients. Since then, the African Union has not issued any instructions or regulations concerning improving DRLs used in radiology across Africa. South Africa, Nigeria, Sudan, Kenya, Egypt, and Cameroon are just a few African countries that have endeavoured to establish their own DRL standards for the future. 

In Table 5, we can see that certain African countries have established DRLs for specific CT procedures. Not all Asian countries, like certain African ones, have fully capitalized on DRL’s contributions to optimizing patient dosing. For example, in the Russian Federation, the possibility of developing DRLs for the public was slowed down by the country’s considerable regional diversity and high costs [30]. To prevent clinically inappropriate patient doses of radiation, however, certain Asian countries are working to establish DRLs connected to CT of popular operations. 

**Table 2:** International DRLs of some countries for a few CT exams (CTDI = mGy, DLP = mGy*cm).

Country	Descriptor	Abdomen	Chest *	Chest **	Pelvic	C-Spine	Head
South India [31]	CTDI_v_	12	10	-	-	-	47
	DLP	550	445	-	-	-	1041
Puducherry, India [32]	CTDI_v_	16	12	-	-	-	32
	DLP	482	456	-	-	-	925
Kerala, India [33]	CTDI_v_	9	5	-	-	-	27
	DLP	319	164	-	-	-	620
IAEA CRPR [34]	CTDI_w_	10.9	9.5	-	-	-	47
	DLP	696	447	-	-	-	527
Jordan [35]	CTDI_w_	17.9	-	-	-	-	-
	DLP	929	-	-	-	-	-
EC [36]	CTDI_v_	13–35	10	10	-	-	60
	DLP	460–1200	400	400	450–650	400–600	1000
USA [4]	CTDI_v_	-	12	13	-	28	56
	DLP	-	443	469	-	562	962
Singapore [37]	CTDI_w_	11	9	-	-	-	41
	DLP	437	226	-	-	-	718
Japan [38]	CTDI_v_	-	15	15	-	-	85
	DLP	-	550	550	-	-	1350
Canada [39]	CTDI_v_	-	14	14	-	-	82
	DLP	-	521	521	-	-	1302
UK [4]	CTDI_v_	14	12	12	-	21	60
	DLP	910	610	610	-	440	970
Australia [4]	CTDI_v_	-	15	15	-	-	60
	DLP	-	450	450	-	-	1000
Korea [40]	CTDI_v_	10.58	-	7.3	-	17.89	63.7
	DLP	1511.41	-	297.05	-	434.04	1119.4
Ireland [41]	CTDI_v_	13	9	9	-	19	58
	DLP	1120	390	390	570	420	940
Syria [42]	CTDI_v_	24.1	22	30.5	27.5	-	60.7
	DLP	721	520	133	542	-	793
Turkey [42]	CTDI_v_	13.3	11.6	11.3	19.4	-	66.4
	DLP	204	289	283	421	-	810

Chest * = non-contrast Scan, Chest ** = Contrast Scan.

**Table 3:** The National Diagnostic Reference Level (NDRL) for CT as it is currently used in a few different Indian states (CTDI = mGy, DLP = mGy*cm).

State	Descriptor	Head	Neck	Chest	Abdomen	Pelvic	AP	C-Spine
South India [31]	CTDI_v_	47	10	-	12	-	-	-
	DLP	1041	445	-	550	-	-	-
Puducherry, India [32]	CTDI_v_	32	12	-	16	-	-	-
	DLP	925	456	-	482	-	-	-
Kerala, India [33]	CTDI_v_	27	5	-	9	-	-	-
	DLP	620	164	-	319	-	-	-

**Table 4:** Some European Union member states’ current national diagnostic reference level (NDRL) for CT [36]. (CTDI = mGy, DLP = mGy*cm).

Country	Descriptor	Abdomen	Chest	Neck	Pelvic	C-Spine	AP	Head
EC	CTDIv	13–35	10	-	-	-	-	60
	DLP	460–1200	400	-	450–650	400–600	-	1000
Switzerland	CTDIv	-	10	20	20	30	15	65
	DLP	-	400	500	500	600	650	1000
Norway	CTDIv	18	15	20	-	20	-	70
	DLP	800	400	-	-	400	-	1000
Slovenia	CTDIv	17	15	-	-	-	-	-
	DLP	555	475	-	-	-	-	-
UK	CTDIv	14	12	-	-	28	13	60
	DLP	560	610	-	-	600	510	970
Germany	CTDIv	-	-	-	-	-	-	-
	DLP	900	400	-	450	-	-	900
France	CTDIv	-	15	-	-	-	-	65
	DLP	-	475	-	-	-	-	1050
Luxembourg	CTDIv	-	-	-	-	-	-	-
	DLP	-	270	440	-	440	-	1000
Sweden	CTDIv	25	20	-	-	-	-	75
	DLP	-	600	-	-	-	-	1200

AP = Abdomen-Pelvis.

**Table 5:** DRLs benchmarks of some African countries were established for a few CT procedures (CTDI = mGy, DLP = mGy*cm).

Exam	Descriptor	Abdomen	Chest	Chest HR	Pelvic	LS	Head	AP
Nigeria [43]	CTDI_v_	15 *	17	-	-	-	61	20
	DLP	757 *	735	-	-	-	1310	1486
Sudan [44]	CTDI_v_	11.6	11.5	-	-	-	65	-
	DLP	437	327	-	-	-	758	-
Kenya [44]	CTDI_w_	20	19	-	21	20	61	18 **
	DLP	1842	895	-	1928	712	1612	1182 **
Cameroon [45]	CTDI_v_	-	52	-	-	25	-	15
	DLP	-	1151	-	-	769	-	716
Morocco [46]	CTDI_v_	-	-	-	-	-	-	-
	DLP	-	-	-	-	-	1408	-
Egypt [47]	CTDI_v_	31	22	22	-	-	30	31
	DLP	1425	420	420	-	-	1360	1325
Ghana [48]	CTDI_w_	35	30	35	35	35	-	-
	DLP	780	650	280	570	780	-	-
Tanzania [49]	CTDI_w_	35	30	35	35	35	60	-
	DLP	780	650	280	570	780	1050	-
Algeria [50]	CTDI_v_	25	-	-	-	35	50	-
	DLP	-	-	-	-	-	-	-
South Africa [51]	CTDI_v_	-	32	-	-	-	32	7
	DLP	-	593	-	-	-	767	386
Cote d’Ivoire [34]	CTDI_v_	-	-	-	-	-	50.9	-
	DLP	-	-	-	-	-	982.879	-
Tunisia [52]	CTDI_v_	-	-	-	25.4	-	24.3	-
	DLP	-	-	-	599	-	874	-

LS = Lumber Spine * = Non-Contrast, ** = Contrast.

The country’s DRLs are shown in Table 6. Highly reproducible and easily measurable dosage measures are required to establish NDRL using a typical man or phantom. DRLs must be adjusted for each country because of variations in patient size and weight and the medical technology and treatment regimens in common usage. 

However, image quality needed for clinical purposes should not be sacrificed while establishing the NDRL. DRL can be used to monitor patient doses and flag those that are significantly above the norm. Evaluating the predicted clinical image quality and DRL is important whenever testing techniques are altered. DRL is not a cutoff point between acceptable and unacceptable methods of radiologic diagnosis. Unlike the dosage limit, DRLs can be lowered if necessary. DRL is unrelated to an individual’s vulnerability. The dose received by an individual patient may exceed the DRL because it is proportional to the patient’s size and weight. For the national review of the previous DRL to be applied using aggregate national radiological dose data, a new local DRL must be approved whenever there is a change in the facility’s equipment or practises. Processes in healthcare institutions should be DRL-compliant without compromising image quality. 

### 1.15. Defining Diagnostic Reference Levels (DRLs)

Diagnostic Reference Levels (DRLs) are tools used in medical imaging to optimize patient radiation doses. They are defined as dose levels for typical examinations for groups of standard-sized patients or standard phantoms for broadly defined types of equipment. DRLs are not dose limits but serve as guidelines to identify unusually high radiation doses that may require investigation.

The primary purpose of DRLs is to promote the optimization of patient protection in medical imaging by

1. Identifying facilities or procedures where doses are unusually high.
2. Encouraging facilities to review their protocols and equipment performance.
3. Providing a benchmark for comparing practices across different facilities.

DRLs are typically established by professional medical bodies or regulatory authorities based on surveys of patient doses from a wide range of healthcare facilities. They are usually set at the 75th percentile of the observed dose distribution for a particular examination.

In optimizing radiation protection, DRLs play a crucial role by helping to balance the need for adequate image quality with the principle of keeping radiation doses as low as reasonably achievable (ALARA). They provide a practical mechanism for promoting continuous improvement in dose management without compromising diagnostic efficacy. The concept of DRLs was first introduced by the International Commission on Radiological Protection (ICRP) [59].

Once sufficient data (typically 20–50 patients per examination) with appropriate dose quantities are collected, easily measurable radiation metrics can be applied to the imaging modality. These metrics are crucial in determining the ionizing radiation dose quantity used for clinical imaging tasks. Therefore, it is essential to have a clear and defined goal for Diagnostic Reference Level (DRL) values in the context of nominated clinical imaging procedures. Researchers and radiologists should carefully consider regional DRL metrics to make informed judgments.

Each examination’s median dose distribution records are highly significant in establishing DRL quantities for each facility. Outliers, which are extreme values, do not contribute significantly to DRL values, as they have minimal impact on the median dose distribution.

Setting DRLs at the third quartile (75th percentile) assures that the mean dose scattering remains below the upper-end distribution, even if there is no scientific evidence to warrant doing so. Therefore, when considering a sizable cross-section of facilities, it makes sense to establish the DRL value at the 75th percentile of the national distribution. However, if the National DRL (NDRL) is set at the 90th percentile, then 90% of the facilities in the survey will have doses lower than or equal to the NDRL, while 10% will have doses higher than the NDRL. 

DRL values are not fixed; they must be revised regularly to account for technological developments and shifts in clinical practice.

Due to its relatively high radiation dose to patients, CT scanning requires careful optimization. DRL quantities are excellent benchmarks for monitoring good practices and helping optimize patient doses while maintaining the desired image quality. Healthcare facilities should strive to achieve and maintain DRL median values or keep doses below the NDRL values for their patients. Setting national DRLs can be accomplished through automated data collection methods or by utilizing the country’s median values of DRL.

DRL values are crucial for ensuring radiation’s safe and effective use in clinical imaging. They provide a means to monitor and optimize patient doses while maintaining high-quality diagnostic images. Regular updates and adherence to established DRLs contribute to improved healthcare practices and patient safety.

### 1.16. Use of DRL Levels

Recent studies have shown that the implementation of DRLs can lead to significant reductions in patient doses without compromising image quality [12]. CT imaging diagnostic reference levels (DRLs) have numerous functions. In order to take remedial action in facilities where examination doses are over the 75th percentile of the mean or median dose distribution, certain countries have developed DRL values. However, it was pointed out that it may be misleading to just look at dosages that are greater than the 75th percentile, as scenarios where doses are below this threshold may also require optimization. To account for this, a new standard called Achievable Dose (AD) has been implemented. 

With today’s technology and well-crafted protocols, we may achieve an Achievable Dosage (AD) that optimises the dose given to each individual patient such that they receive the median or mean amount of radiation. 75 percent of facilities should have dosages at or below the DRL, according to the national standard that is often set at the 75th percentile. Recent studies suggest that even the 75% of facilities that meet the existing DRLs can benefit from optimization by adding A.D. and size-specific dose estimation. This method is useful for reducing and optimising radiation exposure in a wide variety of settings. 

Quality can also be increased through using the DRR (Diagnostic Reference Range) idea. DRR helps keep things in check so that patients are not overexposed to radiation while still receiving adequate clinical image quality. 

Several factors contribute to the importance of dose reference levels (DRLs) for a country, including the need to control dose dispersion, promote and extend good practises, and set up rigorous supervision to prevent excessive radiation exposure. It is critical to review and evaluate examination techniques and equipment performance if DRLs are routinely exceeded in clinical circumstances. When clinical assessments are above the established DRLs, further investigation and optimization of patient dosages are required, followed by periodic reviews. Clinical image quality expectations must be evaluated whenever examination techniques are altered. The primary objective of setting DRLs is to optimize patient dose without compromising the quality of clinical images. It emphasizes the importance of maintaining appropriate radiation levels tailored to each patient’s needs while ensuring their safety and well-being during diagnostic procedures.

Image quality and radiation dose are two essential aspects of computed tomography (CT) imaging that must be carefully balanced to ensure optimal patient care and safety.

### 1.17. Relationship between Image Quality and Radiation Dose

The relationship between image quality and radiation dose in CT is a crucial consideration in medical imaging. It involves finding the right balance between obtaining clear, diagnostically valuable images and minimizing radiation exposure to the patient. Advanced reconstruction algorithms have been shown to maintain image quality while allowing for substantial dose reductions [60].

1. Trade-off: There exists a trade-off between image quality and radiation dose. Increasing image quality typically requires a higher radiation dose to achieve sharper, more detailed images. On the other hand, reducing radiation dose may result in lower image quality with potential loss of diagnostic information. Finding the optimal balance between these two factors is essential for providing accurate diagnoses while ensuring patient safety.

2. Dose Optimization: Radiologists and radiologic technologists follow the ALARA principle (As Low As Reasonably Achievable) to optimize radiation dose. This principle aims to use the lowest possible radiation dose, allowing for sufficient image quality to make accurate clinical decisions. By employing advanced imaging techniques, dose modulation, and iterative reconstruction algorithms, healthcare professionals can achieve this goal.

3. Clinical Indications: The appropriate radiation dose and image quality depend on the specific clinical indications for the CT scan. A balance must be struck for routine diagnostic exams to minimize radiation exposure without compromising diagnostic accuracy. However, higher image quality may be necessary for complex or critical cases to detect subtle abnormalities, even if it means accepting a slightly higher radiation dose.

4. Patient-Specific Factors: The relationship between image quality and radiation dose also considers patient-specific factors. For instance, paediatric patients are more sensitive to radiation, and special protocols are used to reduce doses while maintaining image quality. Similarly, adult patients’ size and body habitus may influence the selection of appropriate scanning parameters.

5. Technological Advancements: Continuous advancements in CT technology significantly manage the relationship between image quality and radiation dose. Improved hardware and software, such as dose-modulation algorithms and iterative reconstruction techniques, enable the acquisition of high-quality images with lower radiation doses.

The relationship between image quality and radiation dose in CT imaging is delicate. Striving for optimal image quality with the lowest possible radiation dose is essential for providing accurate diagnoses and safeguarding the well-being of patients. This is achieved by applying appropriate scanning protocols, state-of-the-art technology, and adherence to radiation safety principles.

## 2. Conclusions

DRLs are a valuable tool for optimizing medical examinations when set at the 75th percentile of any healthcare facility’s median patient dose distribution. The ultimate goal of achieving excellent image quality is to obtain accurate diagnostic information. At a national level, DRLs reduce dose variations, harmonize and expand good practices, narrow the dispersion of radiation doses, promote standardized diagnostic achievement, and establish systematic oversight to prevent excessive radiation exposure.

The responsibility for developing and implementing DRL concepts lies with the authorized regulatory body in each facility. Professionals must approve DRL metrics data collection to establish DRL values and optimize doses. Optimization actions involve investigating local practices, comparing them against DRL values, assessing equipment, operator, and patient-related factors for compliance, employing dose reduction software tailored for CT imaging, reviewing examination protocols, defining image quality criteria, and providing appropriate training to CT professionals.

When optimizing patient protection, it is crucial to consider achieving adequate image quality and providing sufficient diagnostic information. DRLs play a pivotal role in guiding healthcare facilities toward maintaining high standards of patient care while ensuring radiation doses are carefully managed and kept at optimal levels.

## Figures and Tables

**Table 1:** Provides an overview of the global community’s dose contribution due to medical exposure to CT.

Approximate CT Dose Contribution to Population (%)	Dose Contribution from CT Procedures
34%	Worldwide population [1]
50%	UK population [2]
50%	American population [4]
60%	European population [5]

**Table 6:** Recently published national DRLs related to common CT procedures in some Asian countries (CTDI = mGy, DLP = mGy*cm).

Country	Descriptor	Abdomen	Chest *	Chest **	Pelvic	C-Spine	Head	AP
Singapore [37]	CTDI_w_	11	9	-	-	-	41	-
	DLP	437	226	-	-	-	718	-
China [53]	CTDI_v_	20	15	-	-	-	60	-
	DLP	790	470	-	-	-	860	-
Russia [30]	CTDI_v_	-	-	-	-	-	-	-
	DLP	780	500	-	880	-	1190	-
Puducherry, India [32]	CTDIv	-	32	12	16	-	CTDI_v_	-
	DLP	-	925	456	482	-	DLP	-
Kerala, India [33]	CTDIv	-	27	5	9	-	CTDI_v_	-
	DLP	-	620	164	319	-	DLP	-
Iran [54]	CTDI_v_	10	10	-	-	-	43	-
	DLP	-	330	-	-	-	700	550
South India [31]	CTDI_v_	12	10	-	-	-	47	-
	DLP	550	445	-	-	-	1041	-
Japan [38]	CTDI_v_	-	15	15	-	-	85	20
	DLP	-	550	550	-	-	1350	1000
Turkey [42]	CTDI_v_	13.3	11.6	11.3	19.4	-	66.4	-
	DLP	204	289	283	421	-	810	-
Korea [40]	CTDI_v_	10.58	-	7.3	-	17.89	63.7	-
	DLP	1511.41	-	297.05	-	434.04	1119.4	-
Syria [42]	CTDI_v_	24.1	22	30.5	27.5	-	60.7	-
	DLP	721	520	133	542	-	793	-
UAE [55]	CTDI_v_	-	-	-	-	-	-	-
	DLP	-	443	-	-	-	871 */1071 **	671 **
Malaysia [54]	CTDI_v_	-	-	-	-	-	-	-
	DLP	450	600	-	730	-	1050	-
Taiwan [56]	CTDI_w_	31	-	-	-	-	72	-
	DLP	680	-	-	-	-	850	-
Saudi Arabia [57]	CTDI_v_	-	6–16	-	-	-	24–95	5–17
	DLP	-	160–579	-	-	-	495–1435	269–996
Indonesia [58]	CTDI_v_	-	-	-	-	-	62.08	-
	DLP	-	-	-	-	-	1371	-

AP = Abdomen-pelvis, Chest * = non-contrast Scan, Chest ** = Contrast Scan.

## Data Availability

Data sharing is not applicable to this article.
